# Perspectives on early childhood development in China: key dimensions and contextual contributions

**DOI:** 10.3389/fpsyg.2024.1370641

**Published:** 2024-08-14

**Authors:** Qianqian Fu, Fangfang Zhao, Jinliang Qin

**Affiliations:** ^1^School of Early Childhood Education, Hangzhou Polytechnic College, Hangzhou, China; ^2^Experimental Teaching and Demonstration Center for Child Development and Rehabilitation, The College of Child Development and Education, Zhejiang Normal University, Hangzhou, China; ^3^School of Early-Childhood Education, Nanjing Xiaozhuang University, Nanjing, China

**Keywords:** early childhood development, focus group discussion, qualitative, contextual contributions, China

## Abstract

**Introduction:**

The recognition of culture and context as pivotal influences on the developmental trajectory of young children has been underscored by numerous developmental theories. Localized knowledge is essential for comprehending cultural universality with specificity for early childhood development (ECD).

**Methods:**

Thirteen focus group discussions were conducted with professionals, caregivers, and teachers from four regions in China. Thematic content analysis was employed to identify patterns and themes, followed by coding to identify more conceptual units of meaning.

**Results:**

The findings reveal distinct culture-based skills across four domains of ECD in China. These highlight a local culture that embraces a comprehensive, dynamic, and staged perspective on the development of young children. This study elucidates the multidimensional impact of the environment on young children’s development, with a focus on children’s behavioral characteristics and temperament traits, ECEC practices, and ECEC beliefs that transcend identity, culture, and the economy.

**Discussion:**

This study contributes to the assessment of ECD for future cultural comparisons and enhances the scientific understanding of the interplay between developmental skills in young children and diverse cultural expectations and backgrounds.

## Introduction

Developmental theories have long recognized the critical role of culture and the environment in shaping early childhood development (ECD). Numerous foundational theories within the field concur that ECD is dynamic and complex and is inextricably linked to its cultural environment (Vygotsky and Cole, 1978; Bronfenbrenner and Morris, 2006). Extensive research has confirmed cross-cultural variability in ECD and the influence of cultural environments on cognitive growth, language skills, attachment processes, social development, and other domains (Greenfield, 2016; Hindman et al., 2016; Mesman et al., 2018; Jukes et al., 2021). Even in the domain of gross motor skills, which is considered to have universal developmental patterns, infants exhibit significant differences in the early acquisition of basic motor skills. For instance, infants in rural Africa master fundamental motor skills earlier and more independently than those in other regions (Karasik et al., 2015). Studies on child-rearing beliefs, customs, and environments offer explanations, noting that traditional African infant care involves intentional teaching and practice of sitting and walking (Kilbride and Kilbride, 1975; Konner, 1977; Super, 1981). In contrast, mothers in the Western and European samples perceived infant behavior with an emphasis on cognitive abilities rather than motor proficiency, suggesting a lack of consensus on the necessity of teaching infants to sit and walk. Critics argue that child development research often relies too heavily on Western, educated, industrialized, rich, and democratic (WEIRD) samples, perpetuating a universalist approach that may not be globally applicable (Henrich et al., 2010; Morelli et al., 2018). An example is Piaget’s theory of cognitive development, which, while significant in education, has been criticized for focusing on development universality and potentially overlooking the critical role of children’s social interactions (Tudge and Winterhoff, 1993).

Given the growing global acknowledgment of the significance of ECD, it is imperative for policies and practices worldwide to ensure appropriate, accurate, and purposeful assessment and evaluation of ECD skills. However, the absence of a foundational model that comprehends the universality and particularity of cross-cultural early childhood development skills raises questions among researchers and policymakers on how to balance international comparability with cultural specificity (McCoy, 2021). Cross-cultural research indicates that the culturally valued attributes of a well-developed child are not captured by many measures of early childhood (Rao et al., 2020). For instance, the East Asia-Pacific Early Child Development Scales (EAP-ECDS) aim to provide culturally relevant assessment tools for the Asia-Pacific region, addressing the gap in existing measures (Rao et al., 2023). The early Human Capability Index (eHCI) is another example of an effort to create contextually appropriate assessments that consider local cultural norms and values (Zhang et al., 2021). Moreover, when “Western” developmental tools are utilized in developing countries, adaptations are often made with little clarity on how these modifications occur or how decisions for new tool items are made (Gladstone et al., 2010). In the context of globalization, it is crucial for us to acquire a more comprehensive understanding of how abilities are perceived and encouraged across various cultures. Additionally, it is essential to integrate culturally based capabilities into ECD programs and assessments.

China is undergoing rapid social, economic, and political transformations, with significant changes occurring in both policies and practices related to early childhood education and care (ECEC). From the early adoption of Piaget’s child development theory, “action theory,” “ecological systems theory,” and the theory of children’s play during the initial stages of China’s ECEC reform in the 1980s to the introduction of “the zone of proximal development,” “the Reggio Emilia approach,” and “project methods” in the late 1990s, these theories have had a substantial impact on the quality assessment of ECEC education in China (Wang, 2004). However, Zhu (2015) argues that the Chinese ECEC quality standards, which draw from the West, do not sufficiently consider the “Chinese cultural context.” For example, the emphasis on individualized education, autonomy, and independence in ECEC evaluation stands in contrast to traditional Chinese Confucian values that prioritize self-restraint, humility, and the suppression of personal desires (Luo et al., 2013).

Recently, the Ministry of Education of the People’s Republic of China implemented the “Guidelines for Quality Assessment of Early Childhood Care and Education” to further refine the quality assessment system (Ministry of Education of the People’s Republic of China, 2022). The most challenging aspect of quality assessment in ECEC lies in how to scientifically collect, process, and assess extensive information on early childhood learning and development (Xin and Le, 2013). In China, apart from the Children’s Development Center of China Scale (CDCC) compiled by Zhang in 1985, there is still a lack of up-to-date child development measurement tools based on local culture (Zhou and Zhang, 1994). The current basis for evaluating the physical and mental development of young children relies on the “Guidelines for the Learning and Development of Children Aged 3–6” (Guidelines) released by the Ministry of Education in 2012. The “Guidelines” describe the learning and development of children in five major areas (health, science, language, arts, and social studies), outline developmental goals for children of different ages, and provide corresponding educational guidance (Qi and Melhuish, 2017). However, experts suggest that the “Guidelines,” which serve as prescriptive recommendations for early childhood learning and development, should not be regarded as standards for measuring children’s development (Huo and Shi, 2013). Therefore, there is an urgent need to establish further evidence based on localized knowledge to define and understand ECD skills. To address these issues, this study aims to explore (1) concepts and ideas related to young children’s development that are perceived as important by Chinese communities and (2) the influence of cultural expectations and contexts on young children’s developmental process. This paper first presents a theoretical framework that integrates context with ECD. The background, process, and selection of the sample population of the research method were then described. Subsequently, it presents our findings through an exploration and analysis of ECD skills within the Chinese cultural context. Finally, in conjunction with the theoretical framework, this study discusses the significance of ECD skills and contextual meaning in China.

## Theoretical framework for the contextual conditioning of the ECD

The child study movement, which developed in the early part of the 20th century, was propelled by humanist, educational, interdisciplinary, and policy-oriented concerns as well as scientific ones. As this movement gradually took root and expanded within the academic community, it coalesced into “developmental psychology,” which was heavily influenced by the experimental paradigm of psychology (Super and Harkness, 1986). However, in recent decades, the limitations of developmental theories and methodologies have faced scrutiny from critics across various fields (Burman, 1994; Morss, 1996; Corsaro, 1997; James and Prout, 1997). Critics have pointed out that traditional developmental theories often overlook the contextual nature of child development, placing excessive emphasis on discovering universal laws of development and assuming that research findings are applicable in any location and historical context (Jensen, 2012). Moreover, child development has been regarded as a normative and linear process. Concepts such as “ability,” proposed by authors such as Galton, Cattell, Binet, and Terman, as measurable human traits, have been widely applied in educational assessment and prediction (Woodhead, 1999). Individual differences in development that exceed certain thresholds, are often labeled “deviations” or “outliers” and tend to be neglected. The epistemological foundations of developmental psychology primarily rest on positivism and post-positivism, with research methods dominated by experiments, surveys, and objective testing aimed at collecting quantifiable data (Hogan, 2005). Qualitative methods are seldom used and are often deemed unreliable. These limitations have led to a neglect of the diversity within and between cultures. These criticisms have prompted researchers to re-examine and explore more context-sensitive and culturally aware theories of child development.

As an interdisciplinary field of scientific inquiry, developmental science should embrace a broad spectrum of assumptions, principles, or rule systems. By the end of the 1980s, the rediscovery of Lev Vygotsky and the ideas of Urie Bronfenbrenner gained acceptance among a multitude of researchers (Hogan, 2005). Vygotsky highlighted the role of social interaction in cognitive development, while Bronfenbrenner introduced the ecological systems theory, positing that a child’s development is influenced by a multilayered environmental system. They emphasized that a child’s development unfolds within a specific social context and historical timeframe. Currently, an increasing number of developmental psychologists are embracing research methods that carry more cultural significance (Super and Harkness, 1986; Rogoff, 1990; Jahoda, 1993; Goodnow et al., 1995; Kagitcibasi, 1996; Greenfield, 1997). Psychologists and anthropologists have developed a variety of theoretical models to understand the pathways through which culture influences child development. Representative models include Vygotsky’s sociocultural historical theory (Vygotsky, 19), Bronfenbrenner’s ecological system model (Bronfenbrenner, 1994), Harkness and Super’s developmental niche model (Harkness and Super, 1994), Weisner’s ecocultural model (Weisner, 2002), Rogoff’s participatory appropriation (Rogoff, 2003), and Worthman’s bioecocultural model of child development (Worthman, 2010). Although these models differ in their theoretical focus and scope, they underscore the importance of social and cultural environments in child development and view children as active learners and adapters within their environments. For instance, Vygotsky focused on cognitive development and social interaction, Bronfenbrenner on multilayered environmental systems, Harkness and Super on cultural adaptation, and Worthman on the integration of biological and cultural factors.

The evolution of developmental psychology has also fostered the growth of research in the fields of cross-cultural psychology and cultural psychology. However, there are differences in research objectives and methodologies between the two methods. Cross-cultural psychology seeks to develop research protocols based on one culture and then apply them to other cultures for cross-cultural comparisons (Berry et al., 1992). In contrast, cultural psychology seeks to create research protocols grounded in the lifestyle and communication patterns of each culture, using interpretive methods such as ethnographic interviews or focus group discussions to comprehend the perspectives of indigenous members (Greenfield, 1997).

**Figure 1:**
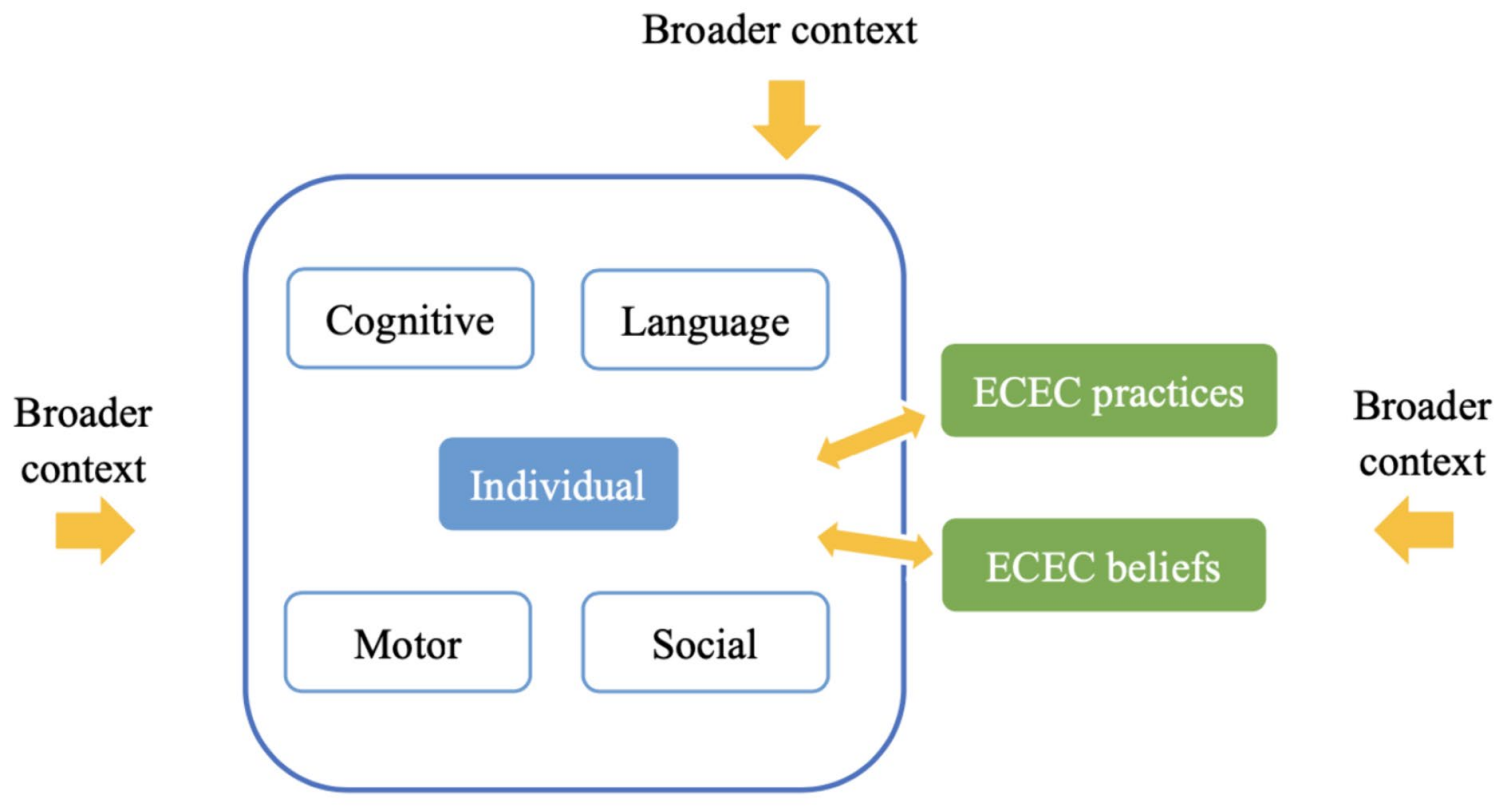
Framework of early childhood development and contextual influences.

Currently, assessments of cultural appropriateness are still dominated by the etic research stance of cross-cultural studies. To overcome this challenge, assessments need to be conducted from the emic standpoint of the research subjects, which in practice requires the experiential and processual experiences of the participants. This study integrates key concepts of cultural psychology into the theoretical framework design and implements research through the interpretive perspective of focus group interviews to examine the concept of ECD in the context of Chinese culture, thereby deepening the culture appropriateness of developmental assessment. Figure 1 presents the theoretical framework of this study, which includes six themes: domains, dimensions, individual characteristics, ECEC practices, ECEC beliefs, and the broader context. (1) Domains and dimensions: Inspired by McCoy’s (2021) framework on the universality and specificity of ECD, we focus on four key domains: cognition, language, motor, and social skills. This approach aligns with the CDCC scale, an assessment tool indigenous to Chinese culture, which similarly categorizes ECD into these four key domains (Zhou and Zhang, 1994). However, we acknowledge the five domains outlined in China’s “Guidelines,” noting the need for clarification on the applicability of the four internationally recognized domains in China. Dimensions refer to the value and interpretation of each ECD skill within a cultural context. Since these values and interpretations are specific to the research findings and require detailed cultural analysis, they are not depicted in Figure 1. (2) Individual characteristics: Positioned at the framework’s core, individual characteristics such as gender and temperament are crucial for understanding developmental processes (Super and Harkness, 1999). Numerous theories emphasize the importance of individual characteristics in development, and the endogenous factors of individual development will inevitably alter the details of the interaction between the individual and the environment (Bronfenbrenner, 1994; Harkness and Super, 1994; Berry et al., 1997). (3) ECEC practices and beliefs: Drawing from the Child Psychology Handbook, these practices and beliefs are defined by cultural analysis units, distinguishing between behavioral routines and symbolic concepts and understandings (Damon et al., 2006). Behavioral aspects encompass the routines of institutionalized family life and social activities, while the symbolic aspects include a series of explicit and implicit concepts and understandings. In this study, we define the behavioral aspects of childrearing culture as ECEC practices and the symbolic aspects as ECEC beliefs. (4) Broader context: The framework recognizes the broader context’s influence on ECD, as emphasized by Bronfenbrenner (1994), Harkness and Super (1994), Weisner (2002), and Worthman (2010). Bronfenbrenner’s ecological systems model provides the most comprehensive and hierarchical perspective on the ecological environment, including macro factors such as society, politics, economy, and culture. Actions, thoughts, and feelings cannot be interpreted in a vacuum; they only make sense through the larger context and cultural understanding. The relationships between the six themes are indicated by the yellow arrows in the figure. The bidirectional arrow in the center represents the interconnectedness of the system, emphasizing causality as reciprocal bi- or multi-directional (←→) or circular (positive and negative feedback loops; Overton, 2013). Although all aspects of the individual and environment exist in mutually influential relationships (Elder, 1998; Molenaar, 2008), considering individual characteristics, ECEC practices and ECEC beliefs are difficult to immediately reflect in their impact on the larger context. Therefore, the outer arrows are unidirectional arrows. Through the interpretation of these key themes, we can more comprehensively understand how developing individuals and broader environment interact and constitute each other in the dynamic process.

## Method

### Research background

This study was conducted over a 6-month period in 2022. Members of the research team came from the fields of child development, psychology, and comparative education, embodying the epistemic value of interdisciplinarity. This is a substudy of the key project of the Chinese National Social Science Foundation, “Research on the Socialization of Early Childhood Care.” Some of the preliminary findings have already been published as doctoral dissertations (Xu, 2022; Zhao, 2023). Thus, the depth of the “phenomenon knowing” emerged through layers of data collection and analysis that both preceded and expanded upon the researchers’ physical time in the field (Kanal and Rottmann, 2021). The prior work included classroom observation, interviews with teachers/principals in early childhood care and education agencies, and child development item testing.

### Research design

The research design is rooted in the theoretical framework, which critiques traditional child development theories for their limited cultural sensitivity despite the emphasis on universality. In response to this limitation, our study embraces an interpretive cultural psychological perspective, aiming to assess ECD in China from a community-based standpoint. This approach prioritizes reflexivity and thorough analysis, favoring interviews and participation observation to capture the intricacies of social phenomena (Hogan, 2005). Interviews are fundamental for identifying and understanding important factors and organizational structures within developmental environments. They transcend the hermeneutic boundaries surrounding objective behaviors, facilitating a profound understanding of the internal meanings of culture (Super and Harkness, 1999). Compared to individual interviews, focus group interviews offer a significant advantage in efficiently gathering rich information in a shorter period (Krueger and Casey, 2014). Moreover, interactions within a group can stimulate more creativity, providing a broader range of ideas and experiences (Vaughn et al., 1996). Based on these considerations, this study selected focus group interviews as the primary method, intending to elicit concepts and perspectives on ECD from local community members in China.

### Setting

Considering the cultural and economic differences between northern and southern China, this study selected four cities, two of which were located in the southern region (Hangzhou and Lishui in Zhejiang Province) and two in the northern region (Taiyuan and Linfen in Shanxi Province). We hoped that four different geographical areas would provide variable perspectives on the topic as a result of differences in urbanity as well as socioeconomic status or dialect. Hangzhou and Taiyuan are the capital cities of Zhejiang Province and Shanxi Province, respectively. Zhejiang Province is located on the eastern coast of China and has a population of more than 65 million according to the 2022 census (Zhejiang Provincial Bureau of Statistics, 2022). Zhejiang is one of the most developed provinces in China, generating 2772.231 billion yuan of GDP in 2010 and ranking 4th among China’s 31 provinces (Yue et al., 2014). Shanxi Province is located in the central region of China and has a population of approximately 36 million people as of 2022 (Shanxi Provincial Bureau of Statistics, 2022). Its *per capita* GDP was 40,557 yuan in 2017, ranking 26th among 31 provinces in China (Zhang et al., 2020).

### Participants

This study employed purposive sampling to ensure that the sample represented key characteristics relevant to the research objectives. A total of 13 focus groups were recruited, comprising one professional group, four teacher groups, and eight caregiver groups, to comprehensively cover the multi-dimensional perspectives of local members on the concept of child development. The selection criteria for the professional group placed special emphasis on interdisciplinary backgrounds, ensuring the participation of experts in the fields of psychology, education, neuroscience, and medicine, thereby enriching the depth and breadth of discussion. Table 1 details the background information of these experts. Teacher focus groups were selected based on years of teaching experience and the age levels of the preschool classes they taught, ensuring the diversity and representativeness of the sample. Six preschool teachers from four different regions were chosen, with 24 members in total, including 2 males and 22 females aged 23 to 48 years. Except for one private preschool in Linfen, the other three preschools were public. While the six preschool teachers from Linfen had junior college degrees, the rest had bachelor’s degrees or above. According to a survey conducted across China involving more than 10,000 families with children aged 0–15 years, 61.6% of families reported that grandparents assisted in raising their children (Guo and Wu, 2023). Grandparents’ involvement in the upbringing of their grandchildren is widespread in China. Consequently, caregiver focus groups were categorized into grandparent and parent focus groups across four areas. Caregiver samples were recommended by the recruited teachers based on criteria such as gender, frequency of interaction with young children, and degree of involvement in child-rearing. Table 2 provides basic information about the local participants from each region, including four grandparent groups (10 males and 12 females, aged 57–68) and four parent focus groups (12 males and 10 females, aged 33–42). Interview invitation letters were sent to the invited individuals, and all agreed to participate. However, due to travel restrictions caused by the COVID-19 pandemic, 2 grandparent participants and 2 parent participants from Hangzhou temporarily canceled their vistis.

**Table 1:** Characteristics of participants in professional focus groups (8 participants in total).

Characteristics		
Profession		
	Psychology	3
	Gifted Education	2
	Special education	1
	Cognitive neuroscience	1
	Pediatrics	1
Sex		
	Female	6
	Male	2
Children of their own		
	Children	6
	No children	2

**Table 2:** Sample matrix showing the number of caregiver participants from 4 areas.

Respondents	No. of participants	Hangzhou	Lishui	Taiyuan	Linfen
Grandfathers	10	3	1	3	3
Grandmothers	12	1	5	3	3
Fathers	12	2	4	3	3
Mothers	10	2	2	3	3
Total	44	8	12	12	12

### Procedure

To ensure ethical research standards, our team strictly adhered to established ethical guidelines. We provided all participants with comprehensive oral and written information about the study, including its objectives, confidentiality commitments, expected benefits, potential risks, and voluntary nature. Each participant received a notification detailing the specific time and location of the focus group discussions. Considering the travel restrictions and control policies during the COVID-19 pandemic in China, we arranged four online synchronous focus groups (professional, Hangzhou teacher, Taiyuan parents, and Linfen parent focus groups) to mitigate the impact on participants’ willingness to participate. The remaining nine focus groups were conducted in meeting rooms provided by local preschools.

Data collection was carried out by two researchers working collaboratively. One researcher followed interview protocol and posed most questions, while the other researcher took detailed handwritten notes. All focus groups were recorded and transcribed to ensure the accuracy and completeness of the data. Transcriptions were meticulously checked against the recordings to ensure that they matched the verbal content and captured non-verbal information (e.g., long pauses, laughter, etc.).

Focus group discussions were structured around two primary research questions: concepts and ideas related to Chinese ECD and the impact of cultural expectations and contexts. The discussion topics were based on the six themes within the research framework, ensuring a systematic and comprehensive approach. In professional focus groups, we explored whether traditional Western categories of ECD (cognitive, language, motor, and social) are applicable to China and discussed culturally appropriate domains or skills. We retained topic guides in expert discussions to inform local focus group discussions. Contributions to the dimensions mainly came from teacher and caregiver focus groups. Participants were asked to provide specific details about skills or behaviors and give examples in different environments (e.g., classrooms, playgrounds, communities, families) to elicit diverse viewpoints. For instance, we asked, “What cognitive/language/motor/social abilities do you think young children should have before the age of 6?” Participants were encouraged to deeply consider the abilities related to the development stages of the children they were familiar with. Our study aimed to explore the concepts of skills in various developmental domains in early childhood. Considering that the differences in skill development between infants and toddlers (0–2 years) might not be as pronounced as those between older children, we decided to focus our research on the children aged 3 to 6 years. Most participants came from preschool classes or families with preschool-aged children, and their insights are crucial for understanding the development of children in this age group. For discussions involving children of other age groups, we distinguished and clarified the differences in the analysis process to ensure the accuracy and reliability of the research results. The second question explored participants’ views on nurturing environments or behaviors related to these developmental skills. We deliberately avoided specifying the four categories of developing individuals, ECEC practices, ECEC beliefs, and broader contextual environments to avoid biasing respondents with a predetermined framework. Recognizing that terms such as cognition and fine motor skills might be too technical, interviewers used more colloquial terms such as *zhili* (intelligence) and *shoubu dongzuo* (hand movements) when addressing caregiver focus groups to aid understanding.

### Data analysis

A thematic content analysis approach was used. First, in our initial coding, we produced a coding framework with the identified themes of the data. This framework was applied for further analysis, after which the codes were modified and added (Brooks et al., 2015). We then conducted a hermeneutic exploration of the latent, interpretive dimension of the material by reading and analyzing longer text passages (Kanal and Rottmann, 2021). For instance, the second level of focused coding enabled us to connect our initial coding of ECD skills, such as *qingshang* (translated as emotional intelligence), but differed from Western definitions to identify a more precise Chinese culture-based meaning. At this stage, we went through the data analysis process and reached a consensus to determine the meaning of the data. Third, we conducted the analysis again and divided the information into manageable parts with numeric data to explore the ECD skills mentioned in the focus group discussions. Finally, the core ECD concepts were identified, and associations with all other cultural expectations and contexts were developed.

Two of the authors reviewed all 13 transcripts and coded the data separately. Nvivo (version 12) was used for software-supported content analysis of the interview material. After each code was created, we compared and discussed any codes that appeared in one person’s analysis but not the others. We subsequently decided on their inclusion to limit the possibility of influencing one another in attributing meanings.

## Results

The results are presented in two main sections. The first section focuses on ECD skills, while the second section examines the influences of cultural characteristics. Within these sections, we further categorize the findings into more specific subsections. The section on ECD outlines the key dimensions within different developmental domains. The section on cultural environmental characteristics delves into individual development, ECEC practices, and ECEC beliefs. Although the broader contextual environment is not separately discussed in the subsections, it permeates the analysis as an interpretive dimension, helping to explain the diversity and complexity of developmental domains, dimensions, nurturing activities, and beliefs.

### ECD domains

All the professionals agreed that there was little difference between the traditional Western and Chinese ECD domains. Although the “Guidelines” significantly impact contemporary Chinese preschool teaching and child development, they do not entirely equate to the criteria for assessing ECD. Consequently, in the subsequent focus group discussions, teachers and caregivers followed the order of cognitive, language, motor, and social skills. Among these four domains, the professionals noted that language and social domains might be the most culturally diverse, but they provided an important discussion of the influence of culture on the motor domain. The focus groups of teachers and caregivers unanimously believed that all ECD domains were crucial, aligning with the philosophy of “holistic development” advocated in Chinese education (Hou and Liu, 2023). In discussions, cognitive, language, and social domains were frequently mentioned, reflecting the special attention teachers and caregivers paid to these aspects during the early nurturing process. Furthermore, the discussions integrated children’s behavioral characteristics and temperament traits, such as courage and competitiveness, into the description of developmental skills. This highlights the importance of balancing holistic development with character cultivation in early childhood education. There were 17 cognitive skills, 16 language skills, 18 motor skills, and 13 social skills in total that were most frequently mentioned by the teacher and caregiver focus groups. Table 3 presents the categories of ECD skills in descending order of frequency of mention and a sample of illustrative statements.

**Table 3:** ECD domains and sample description.

Domain of ECD	Description
**Cognitive**	
1. Quick response	“Whether he or she can accept new things quickly when learning” “Faster than others when learning new things”
2. Memory	“Able to remember long lines” “Just learn it once and remember it”
3. Concentration	“He or She can play alone for more than an hour” “He or She pays more attention and lasts longer than other children”
4. Thinking ability	“He or She has his or her own ideas” “Children should learn how to think logically”
5. Interest	“He or She is curious about everything” “He or She has a strong desire to explore the world”
6. Academic performance	“Intelligence is mathematical ability” “Intelligence of older children can be measured based on their academic performance”
7. Naughty	“Naughty in a reasonable way” “Children who are often criticized by teachers”
8. Visual observation	“Smart children have stronger observational abilities than other children” “Find details from pictures”
9. Draw inferences about other cases from one instance	“If you tell him or her what 4 plus 4 is, he or she can immediately calculate 5 plus 5” “Transfer of knowledge”
10. Knowledge	“He or She knows many things that others do not know” “He or She has learned a lot and understands everything”
11. Imitation	“He or she imitates quickly” “He imitated his sister’s movements and did what she did”
12. Talent	“Be gifted in a certain area, such as having a good sense of music” “Some intelligence is innate”
13. Competitiveness	“Do not give up” “She did not go to sleep until 11 o’clock at night when she learned how to play”
14. Problem solving	“She will seek help from strangers to solve problems” “Be able to solve problems through multiple methods”
15. Distinguishing right from wrong	“Guide him or her to have a correct perspective on right and wrong” “Knowing what to do and what not to do”
16. Sensitivity	“High sensitivity to the outside world” “Babies’ perception of the environment is also a manifestation of intelligence”
17. Application of knowledge	“Apply what he or she has learned to his or her daily life” “Whether he or she can use the new knowledge he or she has learned”
**Language**	
1. Understanding	“He or She can understand parents’ instructions clearly” “Able to understand complex statements”
2. Express ideas	“Able to express his or her needs easily at a young age” “He or She can accurately express what he or she wants to say”
3. Logic	“The logic of the last sentence and the next sentence is smooth” “I do not think his language ability is good because he lacks logic”
4. Vocabulary	“Has a rich vocabulary” “He or She can use conjunctions, like if, because, etc.”
5. Repetition	“He can retell a day, what he did in the morning, what he did at noon, and what he did at night” “Being able to retell one thing from beginning to end”
6. Pronunciation	“The enunciation is clear” “He or She knows the pattern of rhymes”
7. Reading interest	“He or She enjoys reading books” “My grandson likes reading books”
8. Courageous	“Children with good language ability are not afraid to express themselves” “Children currently dare to speak on various occasions”
9. Imitation	“He or she can imitate people or friends” “The language ability of children aged 0 to 3 is in a stage of imitation”
10. Coherent sentence	“Joining sentences together” “Express in an intact way”
11. Speak like an adult	“Chat like talking to adults” “Speaking is more mature than other children”
12.Literary imagination	“In addition to talking about real things, he or she can also talk about imagined or speculated things” “Good at faking a story”
13. Debate	“Able to engage in debates with parents at home” “Debate inspires wisdom”
14. Listening ability	“Able to listen to others quietly” “He or She can sit still when the teacher is giving a lesson”
15. Speak slowly	“There is a child in my class who talks very fast; you do not know what he or she is talking about” “Speaking rate is quite important”
16.Literacy	“He or She is willing to know how this word is written” “He or She can guess the pronunciation of many pictograms”
**Motor (Gross)**	
1. Body coordination	“She likes dancing and has good physical coordination” “She has a strong coordination ability of limbs”
2. Perseverance	“When he or she cannot do a certain movement well, he or she can still persist” “When she was 5 years old, she competed in a 2.6-kilometer race and stuck with it”
3. Balance	“Dribbling needs more dynamic equilibrium skills” “When doing somersaults, his or her body has a good sense of balance”
4. Physical strength	“She usually walks a long way and is not tired at all” “Some children in my class get tired easily when exercising”
5. Courageous	“No matter how high (the sports equipment), he or she dares to climb up and jump down” “He dares not even jump off a very low balance beam”
6. Climbing, running, jumping	“Has a comprehensive ability of running and jumping” “His jumping ability is relatively poor”
7. Speed	“His speed is very fast when running” “Only one or two of the boys in her class could run faster than her”
8. Strength	“She is very strong” “With strong hands, she grabbed the door frame and climbed up”
9. Flexibility	“When turning or crossing obstacles, physical agility is very good” “Dexterous in action”
10. Reaction	“When a ball comes, it depends on reaction to decide the action” “There is a correlation between motor ability and a child’s reaction time”
**Motor (Fine)**	
11. Use scissors	“Cut with clean lines when using scissors” “She can cut circles with scissors”
12. Use chopsticks	“She could use chopsticks when she was 2 years old” “She is good at using adult chopsticks”
13. LEGO	“It took him 10 days to build LEGO bricks with more than 3,000 pieces” “He was very interested in playing LEGO when he was 2 and a half years old”
14. Paper folding	“She can fold the paper neatly in half”
15. Flour food cooking	“She could roll dumpling wrappers when she was 5 years old” “She can knead dough very round”
16. Use a pen	“Most children cannot draw a circle and triangle approximately 4 years old” “Drawing lines; coloring”
17. String beads	“Children’s fine motor ability to string beads varies greatly” “Some children just cannot string the beads into holes”
18. Tear paper	“Some children have been taught many times, but still do not know how to tear open the packaging” “Children with good fine motor skills can tear paper along the crease”
**Social**	
1. Emotional intelligence	“He or she needs to know what to say in various kinds of environments” “He or She can cater to the teacher and talk”
2. Housework/self-care ability	“When entering the kindergarten in the morning, he or she can pack the backpack by himself or herself” “Help grandparents carry things”
3. Politeness	“Say hello to strangers” “Take the initiative to greet others”
4. Emotional regulation	“He or She always loses his or her temper” “Have a stable emotional state”
5. Integration into groups	“Integrate into the class and find friends” “Unity and friendship in the collective”
6. Helpfulness	“Being able to help others” “Help friends when they cry”
7. Popularity	“Whether he or she has many friends” “She is popular in her class”
8. Sharing	“Sometimes we force the older child to share her toys with her younger sister” “Our class has a sharing corner for children to share their favorite toys at home”
9. Adaptation to the environment	“Adapt to the new environment quickly” “Children need to adapt to different environments”
10. Proactive	“Have a bright and cheerful personality, like to communicate with others” “He is willing to take the initiative and present himself”
11. Empathy	“Be considerate of the listener when talking” “She immediately shows concern when I hurt”
12. Love	“Love nature” “Love life, love parents”
13. Cooperation	“Children with good social skills are more willing to participate in collaborative games”

All quotations are verbatim and, to maintain anonymity, are identified by the English letters “H” (Hangzhou), “LS” (Lishui), “T” (Taiyuan), “LF” (Linfen), “F” (professional), “T” (teacher), “G” (grandparent), and “P” (parent). For example, TT means a teacher participant from Taiyuan, and GLF means a grandparent participant from Linfen.

#### Cognitive domain

Local participants generally described intelligence as a comprehensive and diverse ability. When we asked “What do you think an intelligent child aged 0 to 6 years looks like?,” almost all the participants answered by describing the skills of all the ECD domains, among which emotional intelligence was most frequently mentioned. Compared to the traditional Western cognitive skills proposed by professional focus groups (such as mental representation, conservation, and categorization), the consensus of local participants on intelligence is closer to Gardner’s theory of multiple intelligences. For instance, what local participants described as “strong language skills,” “logical mathematical ability,” “good motor skills,” “being sociable,” and “interest and exploration of nature” highly align with the diversity of linguistic intelligence, logical-mathematical intelligence, bodily-kinesthetic intelligence, interpersonal intelligence, and natural intelligence in Gardner’s theory (Gardner, 1993). However, for further analysis, we grouped each ECD skill according to the coding framework.

Table 4 presents a count of the statements the local members in each focus group generated for each cognitive skill. Seventeen cognitive skills were mentioned a total of 125 times in the interviews. The most common cognitive skill mentioned was quick response. The fourth highest ranked cognitive skill, thinking ability, has two meanings: the ability to think independently and the ability to think logically. In addition to “pure” cognitive skills, some behavioral characteristics and temperament traits were considered signs of intelligence by local Chinese members. Some examples of naughty (ranked 7th) and competitiveness (ranked 13th) mentioned by the participants were as follows:

**Table 4:** Number of statements per cognitive skill by group.

Cognitive skills	Hangzhou		Lishui				Taiyuan			Linfen			Total
	HT	HP	HG	LST	LSP	LSG	TT	TP	TG	LFT	LFP	LFG	
1. Quick response	1	2	2	3	1	1	3	0	1	2	2	2	20
2. Memory	1	0	0	2	1	2	0	0	2	1	3	2	14
3. Concentration	1	0	0	0	2	2	3	1	1	0	2	0	12
4. Thinking ability	1	0	1	0	1	3	0	0	3	1	1	0	11
5. Interest	0	2	2	0	2	0	2	2	0	0	0	0	10
6. Academic performance	0	2	0	0	2	3	1	1	0	1	0	0	10
7. Naughty	3	1	1	1	0	1	0	0	0	1	0	1	9
8. Visual observation	0	0	1	1	0	1	1	0	0	0	2	0	6
9. Draw inferences about other cases from one instance	0	1	0	1	2	0	1	0	1	0	0	0	6
10. Knowledge	0	0	0	2	1	2	0	0	0	0	0	0	5
11. Imitation	0	0	0	1	1	0	0	2	0	0	1	0	5
12. Talent	2	0	1	0	0	0	0	0	1	0	0	0	4
13. Competitiveness	0	0	0	0	1	0	1	0	1	0	0	1	4
14. Problem solving	1	0	0	1	0	0	1	0	0	0	0	0	3
15. Know right from wrong	0	0	0	1	1	0	1	0	0	0	0	0	3
16. Sensitivity	0	1	0	0	0	0	0	1	0	0	0	0	2
17. Application of knowledge	0	0	0	1	1	0	0	0	0	0	0	0	2
Total	10	9	8	14	16	15	14	7	10	6	11	6	126

“I’ve found that smart kids, particularly boys, often exhibit more mischievous and energetic behavior” (HT).

“Intelligence is the ability to resist defeat and be competitive. My granddaughter spotted a toy at the square, and upon returning home, I purchased one for her. She stayed up until 11 pm, eagerly learning how to play with it” (TG).

The 9th-ranked cognitive skill, *Juyi fansan,* is an idiom that comes from the Analects of Confucius and is translated as “draw inference about other cases from one instance.” Participants from five focus groups considered it an important sign of intelligence, which is similar to the concept of learning transfer in Western psychology:

“A child’s ability to *juyi fansan*, if you tell him or her what 4 plus 4 is, he or she can immediately calculate 5 plus 5” (LSP).

In addition, local participants considered certain social cognitive ability manifestations of intelligence, such as *dongshifei* (distinguishing right from wrong), which is similar to moral development in Western developmental theories. This was a topic discussed by teachers from Lishui, parents from Lishui, and teachers from Taiyuan.

#### Language domain

Local participants expressed a keen interest in language and discussed the interconnections among language, cognition, and social skills. In essence, they believed that language was a significant manifestation of cognitive ability and was closely linked to social ability. They believed that language was not only a significant manifestation of cognitive ability but also closely linked to social ability. This view aligns with perspectives in Western developmental theories, such as Vygotsky’s sociocultural theory, which emphasizes the central role of language in cognitive and social development. Vygotsky proposed that through language, children can communicate with others and internalize cultural knowledge, which is consistent with local participants’ understanding of language importance (Vygotsky and Cole, 1978).

Table 5 illustrates the frequencies at which each language skill was mentioned in each focus group, totaling 105 mentions for the 16 language skills. Understanding, defined as grasping the idea of adult instructions and simple concepts, is regarded as the most crucial language skill in early childhood. The second-ranked skill, expressing ideas, pertained to the ability of young children to accurately convey their thoughts and needs. Logic skill, which ranked third, was considered not only a cognitive skill but also a vital language skill that signifies the ability to comprehend the logical relationships of sentences and express ideas logically. Chomsky’s theory of generative grammar holds a prominent position in Western psychology. This theory posits that children’s language ability includes not only the mastery of vocabulary and grammar but also the capacity to generate and understand complex sentences (Chomsky, 2014). This is closely related to the logical skills mentioned by local participants. The seventh-ranked language skill, reading interest, which was mentioned by both the professional group and local participants, reflected a perspective on the dynamic measurement of language skills. As one professional explained,

**Table 5:** Number of statements per language skill by group.

Language skills	Hangzhou		Lishui				Taiyuan			Linfen			Total
	HT	HP	HG	LST	LSP	LSG	TT	TP	TG	LFT	LFP	LFG	
1. Understanding	2	2	0	1	1	4	2	1	3	0	0	0	16
2. Express ideas	2	0	0	0	2	1	2	1	1	1	2	0	12
3. Logic	3	1	2	1	0	1	1	0	0	0	0	0	9
4. Vocabulary	2	0	0	0	1	1	2	0	0	0	1	2	9
5. Repetition	1	0	0	0	1	0	1	1	0	2	2	0	8
6. Pronunciation	2	0	0	1	1	1	0	2	0	0	0	0	7
7. Reading interest	2	0	0	0	1	1	1	0	0	0	1	1	7
8. Courageous	1	0	2	1	0	0	2	0	0	0	0	0	6
9. Imitative ability	0	0	0	0	1	0	0	3	0	0	1	1	6
10. Coherent sentence	0	1	0	0	1	0	1	1	0	0	0	1	5
11. Speak like an adult	1	1	0	0	1	0	0	1	0	0	0	1	5
12. Literary imagination	2	0	0	0	0	0	0	0	1	0	1	0	4
13. Debate	1	0	2	0	0	0	0	0	0	0	0	0	3
14.Listening ability	2	0	0	0	0	1	0	0	0	0	0	0	3
15. Speak slowly	0	0	0	1	1	0	0	0	0	1	0	0	3
16. Literacy	1	0	0	0	1	0	0	0	0	0	0	0	2
Total	22	5	6	5	12	10	12	10	5	4	8	6	105

“In fact, many language assessments today may place more emphasis on static measurements, such as literacy and grammar. However, young children’s grammar development occurs rapidly. Therefore, I believe it is more beneficial to examine their reading habits or the overall family reading environment. This approach is more likely to predict their future language development levels from a dynamic or long-term perspective” (F).

Similar to the cognitive domain, concepts related to behavioral characteristics and temperament traits, such as *dadan* (courageous, ranked 8th), were revisited. Participants noted the impact of *dadan* on language expression:

“Children who are good at language expression are definitely more confident and dare to express themselves. When they feel the need to use the toilet or do something, they dare to express their needs to the teacher” (TT).

Five focus groups mentioned *jianghua chengshu* (speaking like an adult, ranked 11th). In local culture, the ability to express oneself and understand language better than others is considered an embodiment of good language skills. In the Hangzhou teachers’ and grandparents’ focus groups, the participants coincidentally talked about the language skill of debate, which is called *duikou* in the Hangzhou dialect. One grandparent stressed that *duikou* reflected language ability:

“*Duikou* is when two people discuss a problem. My granddaughter is attending broadcasting and hosting interest classes…I think this is also (important) in foreign countries’ education. *Duikou* can stimulate one’s wisdom through discussions” (HG).

This debating skill can be compared to debate and critical thinking training in Western education. By integrating these culturally unique themes with Western theoretical frameworks, we can more comprehensively understand local participants’ perspectives on children’s language development.

#### Motor domain

Most of the local participants actively emphasized the importance of motor ability in ECD. This emphasis resonates with Western theories such as Piaget’s theory of motor development, which outlines the progression from simple reflex actions to complex motor skills through environmental interaction (Fischer, 1980). Our findings, however, highlighted the unique cultural aspects of motor skill development valued by the local community. Although the Lishui grandparent focus group, the Linfen teacher focus group, and the Linfen grandparent focus group all discussed gross motor abilities, the content was mostly inconsistent with specific skills and was not reflected in the coding. A total of 18 motor skills (10 gross motor skills and 8 fine motor skills) were mentioned 89 times (Table 6). *Dadan* (courageous), which appeares in the language domain, was also found in the results related to gross motor skills. Interestingly, fine motor skills did not emerge as ability indicators but rather as achievement indicators. As Gottfredson and Saklosfke (2009) claimed, abilities are conceived as causes and achievements as outcomes in the assessment context. Achievements are typically content-specific and culture-specific. In the professional focus group, an expert discussed the relevant policy reasons. This perspective suggested a potential imbalance in the assessment of motor skills that could be influenced by cultural priorities. For example,

**Table 6:** Number of statements per motor skill by group.

Motor skills	Hangzhou		Lishui				Taiyuan			Linfen			Total
	HT	HP	HG	LST	LSP	LSG	TT	TP	TG	LFT	LFP	LFG	
**Gross**													
1. Body coordination	1	0	2	1	2	0	2	2	2	0	0	0	12
2. Perseverance	1	3	0	1	1	0	2	0	0	0	0	0	8
3. Balance	2	1	0	1	0	0	0	1	1	0	0	0	6
4. Physical strength	1	1	0	0	1	0	0	2	0	1	0	0	6
5. Courageous	1	0	0	0	0	0	1	0	1	1	0	1	5
6. Climbing, running, jumping	1	0	0	1	0	0	0	1	1	0	0	0	4
7. Speed	1	1	0	0	1	0	0	0	0	0	0	0	3
8. Strength	0	1	0	1	1	0	0	0	0	0	0	0	3
9. Flexibility	1	0	0	0	0	1	1	0	0	0	0	0	3
10. Reaction	0	0	1	1	0	0	0	0	0	0	0	0	2
**Fine**													
11. Use scissors	1	1	1	1	1	0	1	0	0	0	1	0	7
12. Use chopsticks	0	1	0	1	0	0	0	1	1	1	2	0	7
13. Playing LEGO	0	0	0	0	0	3	1	2	0	0	0	0	6
14. Paper folding	0	1	0	1	0	0	0	2	1	0	0	0	5
15. Flour food cooking	0	0	0	0	0	0	0	0	1	0	3	0	4
16. Use a pen	0	0	1	1	0	0	0	1	0	1	0	0	4
17. String beads	0	1	0	0	0	0	1	0	0	0	0	0	2
18. Tear paper	1	0	0	0	0	0	1	0	0	0	0	0	2
Total	11	11	5	10	7	4	10	12	8	4	6	1	89

“I think in our education system, we tend to focus more on gross motor ability and less on fine motor abilities. For example, fine motor skills are not evaluated as indicators of ability; all ability indicators are placed on gross motor skills” (F).

In the focus group discussions on fine motor skills, one skill related to northern culture, called *zuo mianshi* (flour food cooking, ranked 5th), emerged in the results. This practice not only developes fine motor skills but also reinforces cultural identity and the value of traditional activities. Participants from the Taiyuan and Linfen caregiver focus groups discussed the fine motor skills associated with the *zuo mianshi*:

“They have cooking class in preschool, and she usually makes a bowl of *mao’erduo* (cat-ear shaped, one of Shanxi traditional flour foods) when she comes back from the class” (LFP).

This comparison highlights the unique cultural aspects of motor skill development valued by local participants and reveals both similarities and differences. While Piaget acknowledges motor skills’ role in cognitive development, cultural practices such as *zuo mianshi* illustrate how motor skill development is tied to cultural contexts.

#### Social domain

The frequencies of each social skill mentioned by local FGDs are presented in Table 7. While all the local participants acknowledged the significance of social skills in ECD, discussions on social skills were relatively limited and predominantly focused on the skills of *qingshang* (emotional intelligence, ranked first). The concept of *qingshang* can be related to the Western notion of emotional intelligence as proposed by Salovey and Mayer, (1990), which involves the ability to perceive, understand, and regulate emotions. Both perspectives highlight the importance of understanding and managing emotions to interact effectively with others. However, in the Chinese context, *Qingshang* includes culturally specific practices such as *Chanyan guanse* (examine one’s language and observe his or her countenance) and *yanse* (hint given with the eyes), which were described as adult ways of dealing with the world. These practices are not typically emphasized in Western frameworks. For instance,

**Table 7:** Number of statements per social skill by group.

Social skills	Hangzhou		Lishui				Taiyuan			Linfen			Total
	HT	HP	HG	LST	LSP	LSG	TT	TP	TG	LFT	LFP	LFG	
1. Emotional intelligence	4	2	0	2	1	2	1	4	0	2	2	3	23
2. Housework/self-care ability	0	2	2	0	1	1	1	1	1	0	3	0	12
3. Politeness	1	1	3	1	2	0	1	0	0	0	1	0	10
4. Emotion regulation	2	0	0	0	1	0	2	1	0	1	0	0	7
5. Integration into groups	1	0	2	0	0	1	0	0	1	0	1	0	6
6. Helpfulness	2	1	0	0	0	0	1	0	0	0	0	0	4
7. Popularity	2	0	0	1	1	0	0	0	0	0	0	0	4
8. Sharing	0	0	0	1	1	0	1	1	0	0	0	0	4
9. Adaptation to the environment	0	1	0	0	0	1	0	1	0	0	0	0	3
10. Proactive	0	2	0	0	0	0	0	0	0	0	1	0	3
11. Empathy	2	0	0	1	0	0	0	0	0	0	0	0	3
12. Love	0	0	1	1	0	0	1	0	0	0	0	0	3
13. Cooperation	1	0	0	0	0	0	1	0	0	0	0	0	2
Total	15	9	8	7	7	5	9	8	2	3	8	3	84

“Emotional intelligence means that he or she is *hui shuohua* (so sweet) and he or she can *kanren yanse* (pick up hints given with others’ eyes)” (TP).

The second-ranked social skill, self−/family service, pertains to children’s ability to independently serve themselves and contribute to family responsibilities. This contrasts with Western developmental perspectives, where independence and self-reliance are more strongly encouraged. Sharing (ranked 8th), although discussed in the focus group discussion of both northern and southern cities, was interpreted differently. For example, the Lishui teacher focus group believed that sharing should be based on the child’s willingness; otherwise, sharing becomes a form of moral coercion. In contrast, the Taiyuan teacher focus group viewed reluctance to share as a deficiency in family education. Two distinct perspectives on sharing were presented, as illustrated by the following statements:

“In China, sharing is like *Kongrong Rangli*,^1^ where the older child is expected to accommodate younger siblings. Currently, some children experience a kind of moral coercion. They might say that A does not want to share the toy with me. However, the reality is that the other child obtained the toy first, and the child feels that A must share” (LST).

“I believe that some parents still lack an awareness of instilling the value of sharing in their children. They do not reinforce the concept of sharing learned at school, and as a result, the child may naturally develop a strong sense of ownership, thinking, ‘This toy is mine’. Self-awareness is particularly pronounced” (TT).

The notion of sharing shows distinct cultural interpretations. In the Western context, sharing is often encouraged as part of cooperative play and social learning, fostering a sense of fairness and empathy among peers (Eisenberg and Miller, 1987; Tomasello et al., 2005). In contrast, Chinese interpretations of sharing involve deeper cultural narratives such as *Kongrong Rangli*, which emphasizes familial hierarchy and moral duty. Furthermore, regional differences from China also influence interpretations of sharing. The northern teachers, like those from Taiyuan, view reluctance to share as a deficiency in family education and a sign of selfishness. In contrast, southern teachers, such as those from Lishui, believe that sharing should be based on the child’s willingness, highlighting the importance of considering personal feelings before encouraging sharing.

### Contextual influences

Having outlined the primary ECD skills within each developmental domain, we shift our focus to the dynamic nature of cultural expectations and contexts. Local participants uniformly recognized the significant role played by the social and familial upbringing environment in ECD. This section introduces the findings in three areas: developing individuals, ECEC practices, and ECEC beliefs.

#### Developing individuals

The findings of this study revealed that children’s behavioral characteristics and temperament traits permeated various aspects of local participants’ comprehension of ECD. As noted by Super and Harkness (1986), culturally shaped facets of personality can be deduced from the manners in which they are culturally manifested or “projected” in rituals or belief systems. During the focus group interviews, local participants affirmed the value of young children possessing traits such as liveliness, a willingness to express themselves, sociability, competitiveness, self-confidence, and perseverance. They believed that these individual traits were closely intertwined with ECD domains, particularly in language and social domains. Furthermore, local participants considered the influence of introverted and extroverted personalities on the development of young children. One participant highlighted this issue and compared how introverted personalities are perceived in Chinese and Western cultures:

“The sociality of introverted children normally does not have an advantage in Chinese culture. In foreign countries, some behaviors of introverted children may be considered a sign of genius. When [a child] is alone, he or she may do a lot of thinking and do some fancy things, while our culture (to introverted people) is the opposite” (HP).

In the caregiver FGDs, many participants also expressed concerns about the social skills of introverted children. One grandparent provided the following example:

“My granddaughter also has some shortcomings. She may not be that sociable. She always immerses herself in reading alone, so it’s not that … (another grandparent: she may be a bit weak in communication, it’s okay). I do not think everyone can be perfect” (LSG).

One teacher from Lishui offered a balanced view, acknowledging both the cultural preference for outgoing children and the feasibility of introverted children developing into well-rounded individuals:

“In our culture, we hope that children are outgoing, generous, and confident, but some children are introverted and may not necessarily follow the group. I think it is also feasible that he can get along with himself, arrange his things well, and become a complete individual” (LST).

#### ECEC practices

The second cultural factor we observed was associated with ECEC practices embedded in daily activities that transmit Chinese traditional care customs. Customs for child care can be viewed as behavioral strategies for dealing with children of particular ages within the context of specific environmental constraints (Super and Harkness, 1986). The primary child-rearing practices we identified were intensive rearing as internalized parenting and interest-oriented courses as externalized parenting.

#### Intensive rearing as internalized parenting

Safety was a prominent concern voiced by the majority of local participants, reflecting the prevalence of *jingxi jiaoyang* (intensive rearing) activities in the family life of young children. This heightened focus on safety can be seen as a reflection of Erikson’s theory, particularly during the “autonomy versus shame and doubt” stage, which typically occurs during early childhood, between the ages of 2 and 5 years. Erikson posited that during this stage, children are exploring their newfound independence and developing a sense of autonomy (Erikson, 1994). However, if their attempts at autonomy are met with criticism or failure, they are also susceptible to feelings of shame and doubt. In the context of intensive parenting, where safety is often prioritized, this could stifle children’s autonomy and exploratory abilities, leading them to feel ashamed or doubtful about their capabilities. The ECD outcomes of intensive rearing were weaker gross and fine motor development and reduced resilience to stress. For instance, a teacher from the Tainyuan FGDs shared,

“At our preschool, the use of chopsticks is not allowed for safety reasons. I’ve been in preschool for over 30 years and have not seen anyone use chopsticks before” (TT).

The teachers involved in our research emphasized that ensuring the safety of children was paramount in preschool care and education. The heightened focus on safety concerns led teachers to implement protective measures, such as prompt intervention in conflicts with young children and restrictions on specific outdoor equipment. One professional specializing in special education raised the topic of “crawling” based on his consulting experience when reflecting on the early stages of child development:

“It appears that insufficient experience with crawling is noticeable (other professionals concurred). This matter is particularly evident in grandparenting. Caregivers may impose hygiene restrictions and have stringent cleanliness requirements. In later stages, tasks such as using chopsticks arise. Grandparents might feel that the child is not eating enough and is procceding to feed him or her” (F).

The potential long-term effects of such intensive rearing practices on children’s development, including their ECD skills and ability to adapt to various environments, warrant further investigation.

#### Interest-oriented courses as externalized parenting

Almost all caregivers (90–100% of cases) mentioned that their children were enrolled in various interest-oriented courses. Many caregivers highlighted that cultivating a diverse range of interests was essential for the *quanmian fazhan* (all-round development) of children. The *Quanmian fazhan* is the overarching goal of Chinese education and encompasses the development of moral, intellectual, physical, and aesthetic aspects and labor activity (Gao, 2018). For instance, one grandparent from Hangzhou emphasized the significance of the *quanmian fazhan* and its connection with different stages of development:

“We must promote balanced and comprehensive development, and only with a broad foundation can we thrive. Children at each stage have specific learning tasks; for instance, preschoolers engage in play, primary school students learn and develop interests, and middle school students focus on study” (HG).

A father from Lishui explained that expectations for children’s development at different stages were influenced by social guidance, stating, “It is possible that our child is still in the preschool age group. If he or she is already in high school or junior high school, we may need to focus on his or her studies. This is influenced by social guidance.” Although caregivers’ reasons for enrolling children in interest-oriented courses varied and included cultivating interests, preparing for exams, and addressing specific developmental areas where children may be lacking, the constant factor was that interest-oriented courses have become an integral part of the modern Chinese child-rearing process. The expansion of prosperous education marketplaces has fuelled forms of consumerism and commodification that challenge traditional social child-rearing norms (Zhang, 2020).

#### ECEC beliefs

Unlike the consistency exhibited by developing individuals and ECEC practices, ECEC beliefs manifested in more diverse forms. These beliefs were influenced by different identities, cross-cultural factors, and the economy and were conveyed through complex interactions among individuals.

#### Divergence in ECEC beliefs crossing identity

The divergence in child-rearing beliefs across identities was primarily observed between teachers and caregivers, between grandparental caregivers and parental caregivers, and among caregivers of different genders. These differences underscore the complexity of child-rearing in the context of evolving cultural values and intergenerational value changes (Bian et al., 2022).

In the teacher FGDs, educators highlighted the challenges arising from caregivers’ expectations, particularly the tension between the scientifically informed concept of child development in preschool and the traditional practice of intensive rearing within families. For instance, a teacher from Linfen shared, “I believe parents intervene too much. Once, a child was dressing near me, and I encouraged him to do it himself. Later, his father (observed through surveillance video) criticized that I did not care if his child could get dressed. I explained to him that we cannot take care of everything for the child.” There were similar examples, such as a teacher in Lishui who mentioned that caregivers might question why their children talked about *si* (death) when they returned home. She explained reasons similar to her childhood experiences:

“They simply do not consider it appropriate to discuss *si*. When I was young and said in front of my elders that the vinegar was *suan sile* (sour to death), my mother would say, ‘Do not talk about *si* during Spring Festival’ (LST).”

In our study, grandparents compared their parenting experiences across generations, among generations of children, and among generations of grandchildren. Grandparents acknowledged significant changes in the way they raised their grandchildren. In comparison to the *fang yang* (free-range parenting) experiences with their children, they tended to *chong ai* (indulge) their grandchildren more.

Interestingly, patterns of involvement in child rearing and education varied between caregivers of different genders. Males and females, intentionally or unintentionally, play opposite roles in family life. For example, a grandfather from Lishui explained,

“His grandmother and I are opposites. I am the ‘left’, and she is the ‘right’. She scolds if the child does not eat cleanly and criticizes if the child does not do well. I told her there’s no need to criticize; even adults cannot do that well” (LSG).”

In China, a common saying used to describe gendered caregivers’ involvement in child rearing is “*Yige chang bailian, yige chang honglian*” (one coaxes, the other coerces). Many grandparents and parents remarked on the different roles they played in the family. According to traditional parenting patterns in China, caregivers believe that this balanced approach helps them avoid excessive discipline or indulgence.

#### Divergence in ECEC beliefs across culture and the economy

Over the past three decades, China has undergone significant reforms, transitioning from a planned to a market economy and experiencing globalization. These changes have led to a more competitive social environment (Chen et al., 2009) and increased integration between Chinese and Western cultures (Guthrie, 2006). This transformation has created a unique cultural intersection, leading to changes in ECEC beliefs. Both parents and educators from the post-1980s and post-1990s eras served as cross-cultural comparators, engaging in discussions about beliefs that influenced the process of child-rearing and education. One prominent theme that emerged from discussions with teachers and grandparents in Lishui was the concept of rules and their role in ECD. One teacher participant highlighted a dichotomy between societal norms and individual liberation:

“The societal norms dictate that we aspire for everyone to adhere to rules, yet there are individuals who, by stepping outside these norms, experience a more liberated and self-oriented existence. As educators, our desire is undoubtedly to steer children toward compliance with rules, but paradoxically, those who deviate from established norms often gain more. Thus, I frequently find myself grappling with this inherent contradiction” (LST).

On the other hand, grandparents voiced apprehension about children being excessively obedient to rules and expressed concern that these children might be overly honest and susceptible to bullying. For instance, two grandmothers from Lishui employed a derogatory idiom to characterize their grandson/granddaughter as *xungui daoju* (having behavior that appears rather stiff, excessively observant of conventional standards). The viewpoints of teachers and grandparents offer valuable insights into changing beliefs about parenting. Grandparents, raised during the traditional planned economy era with less emphasis on rules and legal structures, worry that rigid rule-following might hinder their grandchildren’s ability to navigate a competitive society. In contrast, teachers strive to balance social norms and individual freedom, fostering an environment that boosts children’s self-esteem and autonomy while respecting societal expectations. These societal changes significantly impact ECEC beliefs.

## Discussion

**Figure 2:**
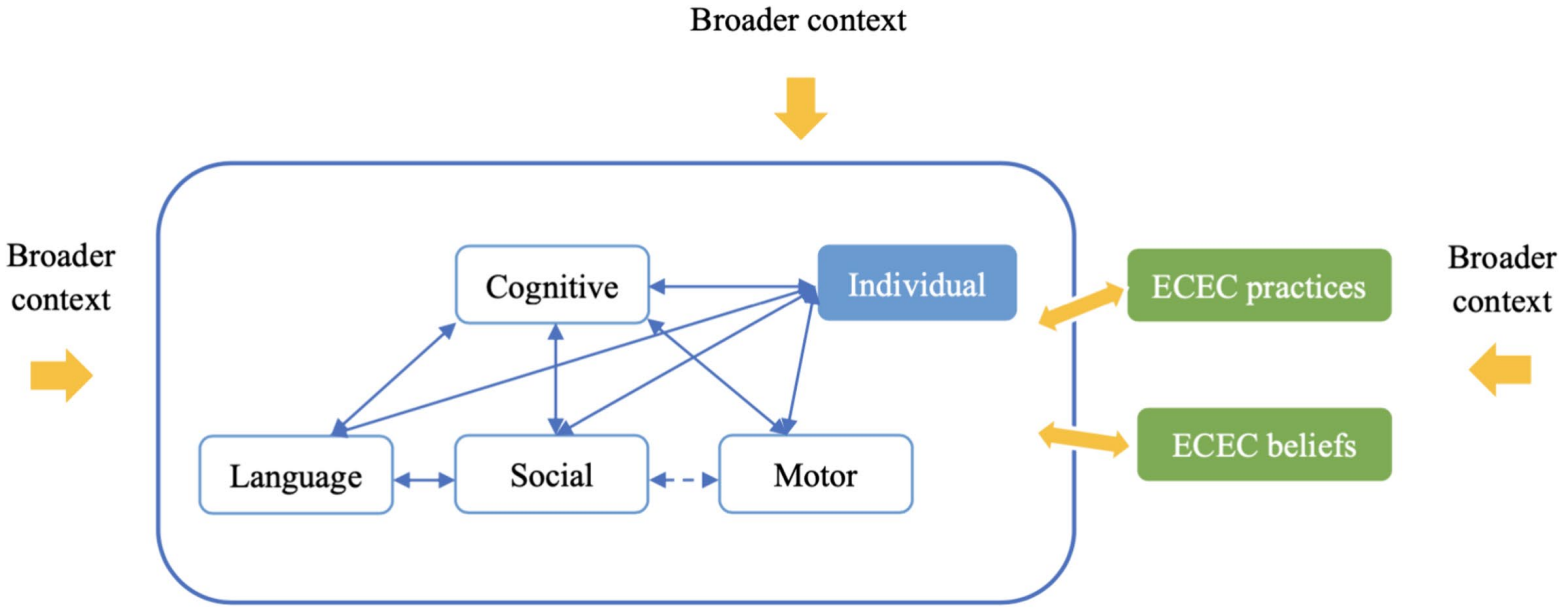
Refined framework of early childhood development and contextual influences.

Cultural research is pivotal for advancing our comprehension of human functioning beyond the assumptions rooted in the cultural backgrounds of most researchers (Rogoff et al., 2017). To our knowledge, this study represents the first application of a phenomenological approach, primarily through focus groups, to offer a contextual and in-depth understanding of the concepts of child development in China. The primary aim of this study was to present a diverse array of themes and languages that provide insights into potential areas for creating new culturally appropriate ECD assessments. Furthermore, it is crucial to contextualize these ECD skills within the broader cultural and social framework because culture profoundly creates and shapes them. As illustrated in Figure 2, we have refined the theoretical framework for ECD in China based on our research findings. The updated framework accentuates the intricate interplay between the cognitive, language, motor, and social developmental domains and the individual’s engagement within the broader cultural and social fabric. It offers a structured approach to examining how cultural norms and values are instrumental in shaping the expectations and practices of ECD in China.

### An integrated, dynamic, and staged perspective on ECD

This study revealed that local culture embraces an integrated, dynamic, and staged perspective on ECD. The concept of “integrated” signifies a comprehensive and interconnected operational view of development. Local participants unanimously agreed that language, motor, and social skills are vital manifestations of a child’s cognitive development. Reflecting this consensus, Figure 2 positions cognitive development at the core, signifying its pivotal role. The solid bidirectional arrows interlinking cognitive development with the other three domains underscore their close relationships, illustrating how these areas are not discrete entities but rather components of a unified developmental process. In the results of the focus group interviews, the connection between the motor and social domains was relatively weak, so we used a dashed arrow to represent this relationship. Since few participants mentioned the connection between motor and language development, we did not use an arrow to link these two domains in the figure. “Dynamic” not only refers to the dynamic nature of ECD (Rao et al., 2020) but also encompasses the expectations of local participants when evaluating developmental skills. This is exemplified by the repeated emphasis on reading habits and interests within the language domain. An ethnographic study of preschool in three cultures elucidated the dynamic nature of developmental goals and educational practices in China (Tobin et al., 2011). The term “stage” does not align with Piaget’s (1964) concept or the Western concept of developmental stages for children (Fischer and Silvern, 1985). Instead, it refers to the social expectations and needs for children’s development guided by Chinese education policies. Local participants expressed distinct requirements for children of various ages, including comprehensive development in preschool, anxiety about preacademic skills during the transition from preschool to primary school, and an emphasis on academic performance in primary school. These staged parenting expectations or beliefs were dynamic and consistent with findings from other studies (Harkness and Super, 1994).

Various culture-specific themes emerged within each developmental domain. The most frequently mentioned cognitive skill by local participants was “quick response.” Zhang’s (2011) study on the personality structure of Chinese children aged 3–12 highlighted that the most crucial manifestation of intellectual traits is “quick response.” Conversely, in Uganda, intelligence is associated with being slow, careful and active (Wober, 1972). Despite the age range of 0–6 years, local participants highly prioritized academic performance, reflecting the traditional emphasis on knowledge in Chinese culture. In the classic Confucian text “Great Learning,” acquiring knowledge is considered the initial step toward perfection (Luo et al., 2013). Furthermore, social intelligence, such as “knowing right from wrong,” is also regarded as an essential component of intelligence. The cross-cultural developmental literature underscores the social aspect of intelligence (Serpell, 1979; Kambalametore et al., 2000). For example, Brazil’s early education prioritizes “values of solidarity, freedom, cooperation and respect” over traditional preacademic skills such as literacy and numeracy (McCoy, 2021).

The deficit approach, which primarily focuses on vocabulary, tends to overlook highly developed language skills (Rogoff et al., 2017), such as narrative fluency, sophisticated use of metaphor, and debate virtuosity (Miller et al., 2005; Averini and Johnson, 2015). In the present study, expressing ideas (ranked 2nd), logic (ranked 3rd), vocabulary (ranked 4th), language organization (ranked 10th), speaking like an adult (ranked 11th), literary imagination (ranked 12th) and debate (ranked 13th) were considered sophisticated language skills by local participants. However, several traditional language assessment items, such as tense and plural forms commonly found in Western tools (e.g., Bayley scales of Infant and Toddler Development), were rarely mentioned. Interestingly, logic was repeatedly emphasized in both the cognitive and language domains. Linguistic researchers have pointed out that English syntax emphasizes formal connection and logical rationality, whereas Chinese syntax places greater emphasis on artistic conception and contextual rationality (Han and Wu, 2014). Language and thinking mutually influence each other. Thus, the characteristics of Chinese thinking lean toward holistic-artistic conceptions but lack emphasis on logical relations.

While fine and gross motor skills are often perceived to be influenced more by medical biological factors than by environmental factors (Weiss et al., 2010), it is noteworthy that local participants and professionals offer a wealth of culturally grounded discussions beyond expectations. The cultural discourse mainly revolves around local participants’ concerns that intensive parenting may hinder the development of both gross and fine motor skills in young children, with limited discussions addressing motor skills. As emphasized by one professional, preschool curriculum indicators typically prioritize gross motor skills, while fine motor skills may not be explicitly outlined or emphasized. Consequently, within the realm of fine motor skills, skills are manifested in specific tasks, such as using scissors, holding a pen, or handling chopsticks, rather than in the form of indicators such as hand-eye coordination, wrist rotation, or dynamic grasping.

Most participants acknowledged emotional intelligence as one of the paramount skills in ECD. A father from Hangzhou highlighted that China is a society characterized by “*renqing shigu*” (a Chinese idiom that signifies the way of the world). Notably, the study revealed that emotional intelligence in Chinese culture has distinct connotations. Salovey and Mayer, (1990) defined emotional intelligence as a subset of social intelligence that encompasses the ability to monitor one’s and others’ feelings, discriminate among them, and use this information to guide one’s thinking and actions. In Chinese culture, however, emotional intelligence is perceived as an adult’s approach to navigating the world. This novel perspective on emotional intelligence across cultures is a noteworthy discovery in the current research.

### ECD and contextual influences

Comprehending and evaluating ECD necessitate a collaborative examination of the developmental environment. Human function is perceived as both an indirect producer and a product of development (Lerner and Busch-Rossnagel, 1981). This study offers a multidimensional explanation of contextual influences on ECD, primarily manifested in developing individuals, ECEC practices, and ECEC beliefs that traverse identity, culture, and the economy.

Individual behavioral characteristics and temperament traits, such as liveliness, sociability, and competitiveness, discussed in the focus groups, not only are crucial for early development skills but also significantly influence ECEC practices and beliefs through a bidirectional dynamic. As depicted in Figure 2, these individual traits wield a strong and direct influence on ECD domains, represented by solid bidirectional arrows that underscore the robust interconnectivity between individual attributes and developmental domains. Moreover, they are themselves significantly shaped and influenced by the broader backdrop of ECEC practices and beliefs, illustrating the intricate and reciprocal dynamics at play within the educational and caregiving landscape. In China, the cultivation of personality traits is considered crucial in the child-rearing process. Fu (2016) found that the affective goals of the Chinese New Curriculum Reform were not purely affective-oriented but were characterized by the strengths and development of virtues (Peterson and Seligman, 2004). Moreover, the influence of children’s behavioral characteristics and temperament on ECEC practices and beliefs is evident. The traditional literature suggests that shy, cautious, and behaviorally restrained children tend to adjust well in Chinese culture (Chen et al., 1995, 1998). However, given the evolving landscape of a new market-oriented environment characterized by both opportunities and competition, individualistic skills such as autonomy, self-expression, and assertiveness have gained increased significance for survival and success (Bian et al., 2022).

ECEC practices and beliefs have diversified due to the intricate interplay of historical shifts, economic factors, and cross-cultural influences. These practices and beliefs are disseminated through individuals and symbols within the environment. The transmission of these influences is not consistent; at times, it occurs through collaborative efforts, while at other times, it leads to exclusion or disharmony, impacting individual development.

Collaborative forces, such as the one-child policy and market-oriented urban ecology, are evident in China’s sociodemographic changes and foster intensive parenting with the “4–2-1 effect” (four grandparents, two parents, and one child) and individualistic values (Zeng and Greenfield, 2015). As one expert noted, “Intensive parenting and free-range parenting are responses to different era backgrounds. In the past, a couple might have had five or six children, which made intensive parenting impractical. Following the implementation of the one-child policy, there was an increased focus on every aspect of a child’s development.” The teacher participants from the four areas all highlighted the pressure from caregivers for “safety.” As mentioned in Tobin et al., (2011) book, the latest generation of parents tends to exhibit excessive anxiety, overemphasizing minor physical health concerns that were previously considered parts of a healthy and joyful childhood. In the current study, specific examples included caregivers imposing strict hygiene standards that limited early crawling exercises (professional focus group), restrictions within preschools on the use of chopsticks for safety reasons (Taiyuan and Linfen teacher focus group), and limitations on certain outdoor equipment (Linfen teacher focus group). Local participants mentioned that intensive rearing may limit the environment needed for the development of early childhood gross and fine motor skills, such as climbing stairs, using chopsticks, and buttoning. In a recent study, Rao et al. (2023) examined secular trends in motor skills development among 4-year-olds in Shanghai (a developed city) and Guizhou (a less developed city) from 2013 to 2017. The results indicated a significant decrease in scores in both regions. However, Rao et al. (2023) explained that a decrease in motor skills may be due to increased screen time and area pollution. This study may provide another compelling explanation for the decline in early childhood motor development in China by attributing this change to intensive rearing, which has been overlooked by other studies.

Traditional strict and critical parenting practices have also evolved. In FGDs, caregivers commonly expressed their aspirations for children to be “healthy” and “happy.” Way et al. (2013) found that the Chinese mothers of seventh graders prioritized the happiness and mental well-being of their children. Ironically, the rapid growth of the market economy and the high-stakes examination system have intensified parental anxieties about childrearing (Zhang, 2020), and present-day preschools fall significantly short of meeting parental demands for education. All the local caregiver participants indicated that their children/grandchildren were involved in more than two interest-oriented courses; some were driven by the children’s interests, while others focused on exam preparation. Studies have shown that shadow education has become an increasingly influential institution that allows individuals to acquire cultural capital, particularly in East Asia (Bray, 2017; Dong and Zhang, 2019). This trend is rooted in traditional Chinese culture, where parents perceive the pursuit of knowledge as a moral virtue (Yang, 2007). While academic pressure may foster ECD in areas such as counting and literacy in the cognitive domain, it can also have adverse psychological effects. A report from the Global Times (2023) indicated that 1 month after the start of school, children’s psychiatric clinics in China were overwhelmed, with a growing trend of patients being increasingly younger in age (China Newsweek, 2021).

Exclusion or disharmony reflects differences in childcare and education beliefs due to the intersection of diverse identities and cultural influences. Differences in identity are primarily manifested in three aspects: teachers’ scientific viewpoints regarding ECD and caregivers’ traditional notions of intensive rearing; intergenerational values in child rearing; and the care and educational perspectives of caregivers of different genders. Although Western parenting theories, such as Baumrind’s (1989) authoritative-authoritarian parenting style and the parental acceptance-rejection paradigm (Rohner et al., 2005), have been extensively applied to Chinese populations (Wang and Chang, 2009), controversy persists regarding whether these Western frameworks fully explain Chinese parenting dynamics (Lu and Chang, 2013). In local focus groups, caregivers discussed the roles they play at home, often adopting “apposite” approaches to strike a balance between avoiding excessive discipline and indulgence. Fu (2016) employed cluster analysis to identify homogeneous family groups and revealed three novel parenting styles that reflect the distinct cultural concepts of rural Western China. Among these, the polarized parenting style was characterized by a combination of rejection and overprotection exhibited by either the mother, father, or both parents. Nevertheless, limited attention has been given to this aspect in studies related to child development or family environments. This finding holds the potential to guide future related research.

According to our findings, differences in parenting and educational beliefs influenced by cultural and economic factors were predominantly observed from the diverse perspectives of local participants regarding terms such as “rules” and “sharing” in the social domain. For instance, a notable disagreement emerged in the discourse on “sharing” between participants from the northern region (i.e., the Tainyuan teacher focus group) and those from the southern region (i.e., the Lishui teacher focus group). Individualist societies emphasize self-direction, competitiveness, and self-gain, whereas collectivist groups emphasize relatedness, harmony, and cohesion (Marcus and Kitayama, 1991; Rao and Stewart, 1999). Given that benevolence is considered a highly prioritized value area in collectivist culture (Schwartz, 1992), northern participants, who were influenced by traditional Chinese culture, believed in encouraging young children to share spontaneously. In contrast, southern participants from China’s developed areas, which are increasingly influenced by Western culture and the new market economy, expressed a stronger inclination toward individualism. This perspective involves considering children’s ownership and respecting their wishes. In addition to sharing, there was discussion about rules. In a planned economy with weak legal infrastructure, harmonious relationships function as an important means of achieving individual goals (Guthrie, 1998; Tamis-LeMonda et al., 2008), and rules are often seen as negotiable or “bypassable.” However, the new Chinese market economy, which has a Western-style legal infrastructure, increasingly demands explicit rules and compliance. This shift may lead to conflicts with the developmental goals of caregivers, especially grandparents.

## Limitations and future directions for research

Despite efforts to recruit a diverse sample, the local participants were mainly from backgrounds with higher socioeconomic status (SES). The findings might exhibit variations if the same study was conducted in remote rural areas, where social change occurs at a slower pace and is perceived differently. Future studies should explore the rural population and examine rural–urban differences. By including a rural population, the results could illuminate how ECD is experienced in distinct ecologies, which would contribute to a more comprehensive understanding of social change and child rearing in modern China. Moreover, in the interviews, intergenerational differences in parenting values concerning child upbringing were predominantly reflected in the perspectives of grandparents and teachers. The study revealed that conflicts often arise between grandparents and parents. When grandparents assume excessive responsibilities and parents are less involved, this can lead to communication problems within the parent–child relationship (Guo and Wu, 2023). In the present study, parents were hesitant to discuss conflicts with grandparents for two main reasons. First, the cultural fabric of Chinese society is deeply influenced by Confucian traditions of filial piety, which underscore children’s obligation to obey and respect their parents (Luo et al., 2013). Second, parents perceive that the active involvement of grandparents in caring for their grandchildren significantly alleviates their work pressure (Liu, 2006). Consequently, they feel grateful to their parents rather than blaming them. Future research could employ self-report questionnaires or individual interviews to more deeply examine the diverse and complex child-rearing ecologies of Chinese families because there is a scarcity of research in this area.

## Conclusion

Understanding ECD requires a contextual approach that acknowledges the multifaceted nature of human development and incorporates an array of environmental factors that collectively shape the growth of children. The field requires enhanced clarity on the manifestation, development, and value of ECD across countries. The successful collection of this evidence hinges on the adoption of a more inclusive approach to studying ECD that dismantles established barriers among different fields, stakeholders, and geographical contexts. By embracing a culturally responsive perspective, this study examines key dimensions within the four main developmental domains of ECD and seeks to comprehend the nuanced interconnections between diverse contextual elements and their impact on ECD.

## Data availability statement

The original contributions presented in the study are included in the article/supplementary material, further inquiries can be directed to the corresponding author.

## Ethics statement

The requirement of ethical approval was waived by Child Development and Rehabilitation Experimental Teaching Demonstration Center at Zhejiang Normal University’s College of Child Development and Education. The studies were conducted in accordance with the local legislation and institutional requirements. The participants provided their written informed consent to participate in this study.

## Author contributions

QF: Writing – original draft, Writing – review & editing. FZ: Writing – original draft, Writing – review & editing. JQ: Writing – original draft, Writing – review & editing.

## Footnotes

^1^(An ancient Chinese story) About a thousand years ago, there was a boy named Kong Rong. One day, his father placed a plate of pears on the table for the boys to eat. Kong Rong was the first to pick a pear, but he picked up the smallest one and let his older brothers and younger brother have the larger ones.
